# Predicting Human Motion Signals Using Modern Deep Learning Techniques and Smartphone Sensors

**DOI:** 10.3390/s21248270

**Published:** 2021-12-10

**Authors:** Taehwan Kim, Jeongho Park, Juwon Lee, Jooyoung Park

**Affiliations:** Department of Control and Instrumentation Engineering, Korea University, 2511 Sejong-ro, Sejong-City 30019, Korea; kteaw0110@korea.ac.kr (T.K.); seanpark0107@korea.ac.kr (J.P.); saero94j@korea.ac.kr (J.L.)

**Keywords:** wearable sensors, human motion, prediction, recurrence plot, Fourier neural operator

## Abstract

The global adoption of smartphone technology affords many conveniences, and not surprisingly, healthcare applications using wearable sensors like smartphones have received much attention. Among the various potential applications and research related to healthcare, recent studies have been conducted on recognizing human activities and characterizing human motions, often with wearable sensors, and with sensor signals that generally operate in the form of time series. In most studies, these sensor signals are used after pre-processing, e.g., by converting them into an image format rather than directly using the sensor signals themselves. Several methods have been used for converting time series data to image formats, such as spectrograms, raw plots, and recurrence plots. In this paper, we deal with the health care task of predicting human motion signals obtained from sensors attached to persons. We convert the motion signals into image formats with the recurrence plot method, and use it as an input into a deep learning model. For predicting subsequent motion signals, we utilize a recently introduced deep learning model combining neural networks and the Fourier transform, the Fourier neural operator. The model can be viewed as a Fourier-transform-based extension of a convolution neural network, and in these experiments, we compare the results of the model to the convolution neural network (CNN) model. The results of the proposed method in this paper show better performance than the results of the CNN model and, furthermore, we confirm that it can be utilized for detecting potential accidental falls more quickly via predicted motion signals.

## 1. Introduction

The world-wide use of smartphones offers not only the convenience of accessing and searching information anywhere, but also various applications to enhance the quality of life. As smartphones have developed, various sensors have been included in smartphones, and a variety of accompanying applications have been introduced. Recently, in addition to basic IMU sensors, various sensors such as photoresistors, fingerprint sensors, and GPS have been added to smartphones, and the performance of the cameras has also improved, so that various studies using these can be conducted. In this paper, we intend to deal with healthcare-related problems utilizing these smartphone sensors. Various research topics of interest in healthcare include analyzing human behavior, and recent studies have been conducted on recognizing and characterizing human motion patterns [1,2,3,4,5,6,7,8,9,10,11]. Various previous studies have examined the recognition or analysis of human motion, and a number of studies have examined recognition of human activities via machine learning [5,6,7,8,9]. Of particular interest is research on aspects of human gait, e.g., while walking and running [12]. Many studies have been conducted on motion analysis using classical to modern machine learning methodologies, such as Gaussian processes [2,3], to the latest deep learning techniques [4,5,6,7,9,10,11]. Wang, J. et al. [2] proposed Gaussian process dynamical models to characterize human motion from captured motion data. Their model was constructed with latent space to represent the dynamics of motion and observation space, and the model showed remarkable results in the latent space. Kim, T. et al. [3], and Kim, J. et al [4] used an accelerometer and a gyro sensor to characterize human movement in latent space. In one study [3] the proposed model could be seen as a combination of the variational autoencoder and a Gaussian process, and the detection of risks such as falling was examined by using the characterized latent space. One particular study [5] showed suitable performance in human activity recognition tasks using magnetic induction and recurrent neural networks, and in another study [7], human activity recognition was achieved by converting signals obtained from triaxial accelerometers into images. These studies sometimes used camera sensors, or sometimes wearable sensors to obtain health signals when attached directly to individuals to analyze and predict movements. Typically, inertial measurement units (IMUs), such as accelerometers and gyroscopes, have been widely used to measure health signals. These sensor signals are generally in the form of time series, and many methods exist for dealing with this data. A spectrogram, which is represented by combining properties of waveforms and spectra, is a widely used method to convert time series data to an image type, as  it visualizes changes in amplitude with time and frequency. Numerous previous studies have used spectrograms for recognizing human activities [13,14,15,16]. In addition to spectrograms, many other techniques exist to render time-series data as image formats. The easiest method to transform sequential data into image data is to image the signal itself, and another technique is to render the three axes of signals obtained from sensors directly into a corresponding RGB format [17]. Zheng, X. et al [17] compared the performance of various signal processing methods such as raw plots and multichannels for human activity recognition. A method of imaging real-valued signals from sensors by dividing them into integers and decimal places has also been introduced [18]. A recurrence plot is another popular method for processing time-series data as images, and some studies have been conducted on recognizing human activities with such recurrence plots [19,20,21,22]. Specifically, recurrence plots can show the global and local contextual features of time-series data, and  through these features, temporal information about the data can be captured.

Recently, notable results have been demonstrated in many fields using deep learning methods. In the field of computer vision, deep learning models that perform better than humans in simple classification problems have been researched. Such methods have also demonstrated excellent performance in studies of topics such as object detection and segmentation tasks, and in even in natural language processing, deep learning models have been used to show results that are almost similar to or superior to human performance. In such studies, the use of convolutional neural networks (CNNs) and fully connected networks have generally shown solid performance in video and image fields, and recurrent networks such as RNN, LSTM [23], and GRU [24] have shown notable results in speech and natural language processing. For example, recent studies have reported suitable performance in speech and natural language processing with CNN [25], and also noteworthy outcomes in video and image fields with the transformer model, which is a similar series of recurrent networks designed for 1D sequence data processing [26,27]. Hence, the use of deep learning models has traversed the boundaries of domains like video and audio, making for a wide range of applications beyond those of specific networks that have shown excellent performance in specific fields. In addition to this expansion of applications of deep learning models, other studies in a different vein have been undertaken. In addition to major research fields using deep learning to determine decision boundaries or to estimate functions through regression, deep learning has recently been used to solve differential equations [28,29,30,31]. Euler’s methods for solving ordinary differential equations (ODEs) are similar to those used by recurrent neural networks such as RNN, LSTM, and GRU [31]. For such reasons, as in [31], studies have been conducted on solving ordinary differential equation with neural networks, and, in [28,29,30], neural networks were used to solve partial differential equations (PDEs). Specifically, the study [30] proposed a method, Fourier neural operator (FNO), to solve the PDE problem by combining neural network architecture and the Fourier transform. The architecture introduced in the study [30] can be understood as a type of CNN, but this method also has the properties of mesh-independence, in contrast to traditional, fundamental CNN. This feature means that when given an input of type N×N rather than the size of the learned input, it works even if the size of the input is an FNO model trained with M×M. Therefore, as long as the model is trained with one resolution, inferences can be made even with other resolutions without retraining. Based on these advantages and features, this method is utilized in this study to predict human motion signals, and we show that this leads to performance superior to that of the CNN architecture through our experimental results.

**Figure 1:**
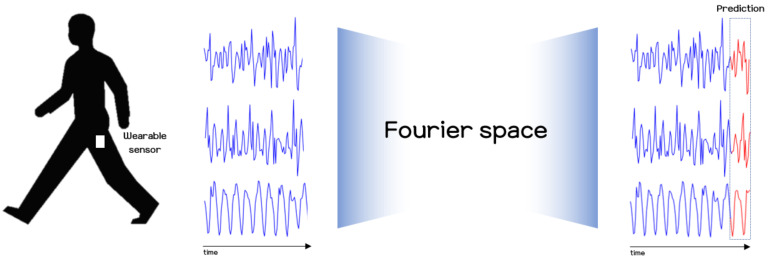
Overview of our proposed method for predicting human motion using wearable sensors and the modern deep learning model.

In this paper, research on human motion analysis was conducted, with the primary objective of presenting a method for predicting human motion signals using modern deep learning techniques and smartphone sensors. Our method relies on the FNO model for its excellent representational capability in the frequency domain, and recurrence plots for their efficiency in handling local and global features. Recurrence plots were originally proposed as a visualization tool for analyzing characteristics of dynamical systems. Recently, many studies have applied these methods to the task of recognizing human activities by making use of the characteristics of recurrence plots, as they are able to capture temporal patterns over small scale and large scale patterns. However, through previous studies [19,20,32,33], it was found that much information loss may occur due to the threshold and step functions in the original recurrence plot formulation. To avoid this problem, some studies used only the distance component to construct recurrence plots [20,32]. Referring to these previous studies, we also use the distance component of the original recurrence plot to convert sensor data into 2D image data, and apply this to the modern deep learning methodology, FNO, to predict a user’s future movements. For more details of the recurrence plot, and how it is used in our experiments to handle sensor signals as a visualization, one may refer to Section 2.1 below. In the recurrence plot, not only small scale patterns but also large scale patterns are revealed, so in order to fully utilize the recurrence plot, we consider use of the FNO model, which can capture the global context using the Fourier space. The overview of our method for estimating sensor signals is sketched out in Figure 1. Using the method presented in this paper, we also explored the possibility of using it for risk detection, such as for falls.

The paper is structured as follows: In Section 1, we briefly explain the rationale for this research and the methods utilized, and introduce related works. In Section 2, as a method for predicting wearable sensor signals, we will introduce the latest deep learning model and the conversion of time series data into a form suitable for application to the model. In the next portion, Section 3, we present the process for acquiring the data used in the experiments, and show the results of the experiments with comparisons. The final Section 4 includes conclusions and discussion of applications for the methods presented in this paper.

## 2. Methods

**Figure 2:**
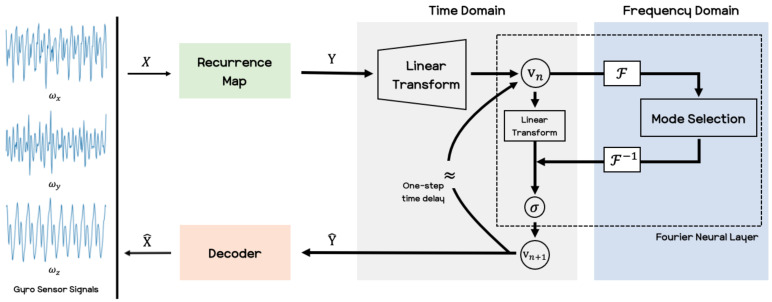
Pipeline schematic of our proposed method for predicting upcoming human motion signals. The linear transform lifts the recurrence plot to a high-dimensional representation or performs a linear combination of the features. A Fourier neural layer composed of a Fourier transform, F, and its inverse, $F^{-1}$, crosses between the time domain and the frequency domain, and it extracts features by removing unnecessary frequencies from the mode selection. Then the output of the Fourier neural layer is given as $v_{n+1}$ which is applied to the sigmoid function, σ.

In this section, we introduce the methods used in this paper. As mentioned in the previous section, we develop an estimation of a user’s activity signals by means of wearable sensors that are built into the user’s smartphone. Among the many kinds of sensors in a smartphone, we utilize the IMU sensor to forecast human motion signals. The acquired raw sensor signals are time-series forms of data, then we transfer the signals to an image format. After converting the data, we use the recently introduced deep neural network known as a Fourier neural operator [30], hereafter referred to as the FNO model, to predict human motion. We consider the recurrence plots [34], which is powerful for converting time-series data to image data. Recurrence plots generally deal with all time steps at once, but we split the time steps with a time window and then use this as the short-term recurrence plot. Figure 2 shows the overall outline of our method used in this paper.

### 2.1. Recurrence Plot

Various studies have recognized and attempted to predict human activities, using not only raw sensor signals themselves via normalization, but also raw sensor signals as transformed images, such as raw plots, multi-channels [17], spectrograms [13,14,15,16], and recurrence plots [19,20,21,22,34]. The raw plots method transforms raw sensor signal data directly to an image, i.e.,  plotted images along axes corresponding to the time and amplitude of the sensor signals. Another transformation, the multichannel method, renders the sensor signals with three axes, i.e., x-, y-, and z-axes, and translates them into to the three color channel components (red, green, blue), in an RGB color format [17]. The spectrogram representation is widely used for dealing with time-series data from speech waveforms in acoustic modeling to IMU sensor data for human motion recognition. It provides a representation of changes in the energy content of a signal as a function of frequency and time [15] and thus shows advantages in classification accuracy and computational complexity [14]. In addition, many other methods exist, but in this paper, the recurrence plot is considered for preprocessing raw sensor signals.

**Recurrence Plot** The recurrence plot (RP) was originally a visualization tool to study complex dynamic systems, which was first proposed in [34]. A recurrence plot expresses information by using the difference between time *i* and time *j* in time series data. It can thus represent time correlation, and based on differences between values at each time step, it is formed with small and large-scale patterns. The small and large patterns provided in a recurrence plot are represented as *texture* and *typology* respectively [34]. These patterns provide information about dynamical systems, and the information is displayed as diagonal lines, dots, vertical lines, and such in the recurrence plots. The recurrence matrix for the recurrence plot is computed as follows when time series data $X=[x_{1},\ldots ,x_{T}]$ is given.

(1) $$Recurrence\left(X\right)=\Theta (\epsilon -||x_{i}-x_{j}||)\;\mathrm{where}\;i,j=1,\ldots ,T\;\mathrm{and}\;x_{i}\in R^{D}$$

Here, ϵ and Θ(·) are, respectively, a threshold value and the unit step function, which yields 0 or 1, depending on the given value. The component ||·|| is a norm, and a proper norm should be selected according to the task. The most commonly used norms are the $L_{1}$ norm, $L_{2}$ norm, and $L_{\infty }$ norm. The general recurrence matrix has a square matrix form, as it represents only one dynamical system. Two typical variants of the recurrence plot can express multivariates, the cross recurrence plot and the joint recurrence plot.

**Cross Recurrence Plot** The cross recurrence plot (CRP) is one representative variant of the recurrence plot, and it is computed by comparing all states occurring in one dynamical system with all states occurring in another dynamical system. In the CRP, the two systems must have the same state dimension, but the length of time does not have to be the same, resulting in a cross recurrence matrix that may not be square. When two systems, $X=[x_{1},\ldots ,x_{T_{x}}]$ and $Y=[y_{1},\ldots ,y_{T_{y}}]$, are given and they are the same dimension, the cross recurrence matrix can be computed as follows.

(2) $$CrossRecurrence\left(X,Y\right)=\Theta (\epsilon -∥x_{i}-y_{j}∥)\;\mathrm{where}\;i=1,\ldots ,T_{x},j=1,\ldots ,T_{y}\;\mathrm{and}\;x_{i},y_{j}\in R^{D}$$

In order to generate proper inputs for machine learning models, in this paper, we follow the basic form of the recurrence plot, Equation (1), and use the $L_{2}$ norm. However, we did not use the threshold, ϵ, or the step function, Θ. In previous studies [19,20,32,33], it has been reported that when using the recurrence plot as the input of the deep neural network model, the thresholds and step functions can be sparse recurrence plots, resulting in information loss. In order to avoid such problems, we follow the strategy [20,32] of calculating recurrence plots without the step function and threshold. That is, we obtained recurrence plots using only the distance component of the recurrence plot.

(3) $$Recurrence\left(X\right)=\left|\right|x_{i}-x_{j}{\left|\right|}_{2}\;\mathrm{where}\;\;i,j=1,\ldots ,T_{w}$$

In the experiments, we use smartphone sensors which are composed of three axes, and by using them, we conduct for predicting human motion signals. Our approach is similar to CRP, which builds its recurrence plots using multiple systems.

### 2.2. Fourier Neural Operator

**Fourier Transform** The Fourier transform is commonly used in image processing to decompose into components corresponding to sine and cosine. Filtering can be applied to an image by using the Fourier transform. For example, when a low-pass filter is applied to an image, the filtered image would appear blurred, with indistinct boundaries. On the other hand, a high-pass filter produces a sharp image with only the edges remaining. The low-frequency component is the portion of small differences in color between pixels, while color differences figure significantly in the high-frequency component. Many studies using Fourier transforms have been conducted on the universal approximation theorem [35], modern deep learning architecture [36], and other problems. Through these previous studies on applying Fourier transforms to neural networks, deep learning has been able to see advances in performance. Among these studies, one recent study directly combined a Fourier space with a neural operator architecture [30]. In this paper, we utilize this architecture to predict human motion signals.

**Figure 3:**
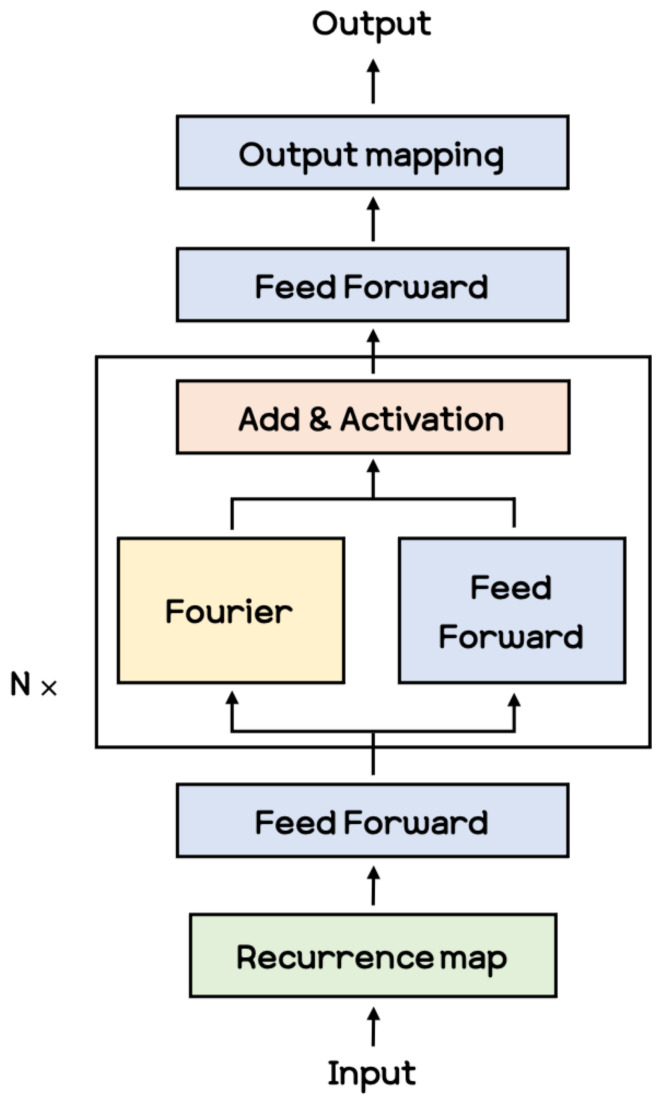
FNO architecture with N Fourier layers.

**Fourier Neural Operator** Within the research on deep neural networks, a new type of neural architecture has been recently introduced, known as neural operators [28,29,30,37]. The architecture proposed in [30] is shown in Figure 3. The Fourier module in this figure performs convolution operations in the frequency domain through the Fourier transform, and then the inverse Fourier transform is applied to revert it to the spatial domain. The Fourier module in the Fourier layer are expressed as Equation (4).

(4) $$F_{n}\left(x\right)=F^{-1}\left(R_{\phi_{n}}\cdot \left(F\left(x\right)\right)\right)\;\mathrm{where}\;\forall x\in D$$

Here, F and $F^{-1}$ are the Fourier transform and its inverse transform, respectively. $R_{\phi }$ is the parameter that functions like a kernel in convolution neural networks.

The Fourier module, $F_{n}\left(x\right)$, receives the high dimensional representation of inputs first and then receives the previous output of the Fourier layer for N-1 times. The Fourier layer consists of the Fourier module and a linear transformation, which can be expressed as Equation (5).

(5) $$v_{n+1}\left(x\right):=\sigma \left(F_{n}\left(v_{n}\left(x\right)\right)+W_{\theta_{n}}v_{n}\left(x\right)\right),\mathrm{where}\;\forall x\in D$$

Here, σ is a non-linear activation function, and in our experiments we used the sigmoid function. We use this Fourier neural operator as a substitute for convolution neural networks. The entire process for training the Fourier neural operator using the recurrence plot data described in Section 2.1 is shown in Procedure 1.
**Procedure 1**: Procedure for Performing Fourier Neural Operator1:Initialize weights θ and ϕ.2:Set parameters of prediction time step and the input time step, $\hat{T}$ and *T*, respectively.3:Get batch RP data for input, *Y*, and target, $Y^{target}$, through Procedure 2.  4:**while** converge **do**5:    $v_{0}={\mathrm{Linear}}_{\theta }\left(y_{t-T:t}\right)$6:    **for** n = 0, …, N−1 **do**                                           ▹ Fourier neural layers7:        $f_{n}=F\left(v_{n}\right)$                                                      ▹ frequency domain8:        Select *k* frequency modes, $f_{n}^{k}$9:        $f_{n}^{k}=R_{\phi_{n}}\cdot f_{n}^{k}$10:        $v_{n}^{F}=F^{-1}\left(f_{n}^{k}\right)$11:        $v_{n}^{S}={\mathrm{Linear}}_{\theta_{n}}\left(v_{n}\right)$                                              ▹ spatial domain12:        $v_{n+1}=\sigma \left(v_{n}^{F}+v_{n}^{S}\right)$13:    **end for**14:    ${\hat{y}}_{t:t+\hat{T}}={\mathrm{Linear}}_{\theta }\left(v_{N}\right)$15:    $L=\mathrm{MSE}(y_{t:t+\hat{T}}^{target},{\hat{y}}_{t:t+\hat{T}})$16:    Update weights, $\left[\theta^{k+1},\phi^{k+1}\right]\leftarrow \left[\theta^{k},\phi^{k}\right]-\alpha L$17:**end while**

### 2.3. Decoder

**Figure 4:**
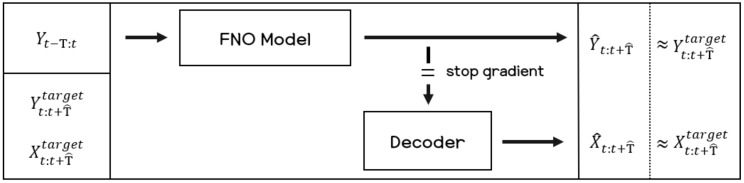
Block diagram for training the proposed model.

In this paper, we use a decoder model to reconstruct the sensor signal from the recurrence plot data. Given the predicted outputs of the FNO model, the decoder model converts the recurrence plot images to the raw sensor signal. The architecture of the decoder used in the experiments is composed of two components, which are a long short-term memory (LSTM) and two fully connected layers. To train the decoder model, both the recurrence plot and raw sensor signals are used. Figure 4 shows the overall process of the proposed method in this paper. For training the FNO model and decoder model, three types of data need to be prepared: the input recurrence plot data as input to the FNO model, *Y*, and the target recurrence plot data that are to be predicted from the FNO model, $Y^{target}$. Lastly, the target sensor signals, $X^{target}$, are to be matched with the outputs predicted from the decoder model.

## 3. Experiments

### 3.1. Data Collection

**Figure 5:**
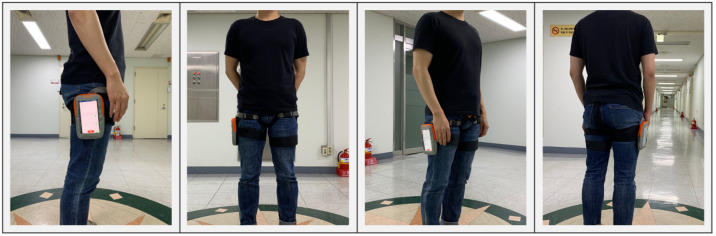
Placement of the smartphone used for data collection.

For the experiments, we use a single smartphone and attached it to the user’s right thigh. We refer to some previous studies [38,39,40,41] and public datasets, such as MobiAct [42] and UC-HAR [18], for the human activity recognition tasks. They used a smartphone and placed it in the trousers pocket to collect built-in sensor signals in the smartphone. We also considered placing the smartphone in the pocket based on the previous studies. When we put it in the trousers pocket and acquired sensor data, we found not only signals from the user’s movement but also noise caused by moving around in the pocket in addition to the user’s movements. For this reason, we fixed the smartphone near the right trousers pocket to obtain only the user’s movement signal without any noise. The location and manner of fixation are shown in Figure 5.

**Figure 6:**
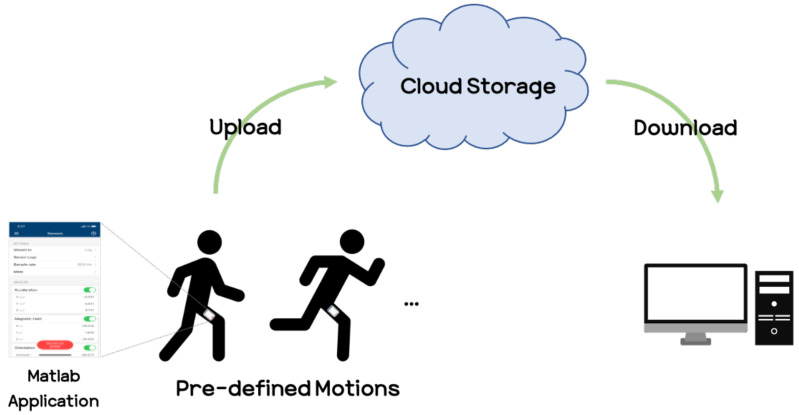
Process for acquiring sensor signals.

The experiments were conducted at the Korea University R&D Center. To collect smartphone sensor signals, we used the Matlab application [43] and the iPhone XS model for detailed smartphone specifications, referring to the manufacturer’s website [44]. A modern smartphone contains many sensors such as an accelerometer, gyroscope, and magnetometer. However, it has been known through previous studies that using the magnetometer should be used with caution due to problems such as direction dependence [45,46] and inefficiency in recognition performance [47,48]. Thus, in our experiments, we considered the use of accelerometers and gyroscopes, and we experimentally confirmed that using the gyroscope yields better performance than the accelerometer. Hence, we choose the gyroscope for acquiring the angular velocity corresponding to the user’s motion. The angular velocity signal was acquired at 30 Hz, which was also experimentally selected between 10 Hz and 100 Hz. To obtain the gyro sensor signals at 30 Hz, we used the Matlab application, which can access the built-in sensors in the smartphone. In our experiments, Matlab was used only for data collection when the experiments were conducted. The data collection procedure consisted of the following: executing the Matlab application on the smartphone, entering the Sensors tab, and after setting the sensors to be used, pressing the start button to acquire sensor signals such as those from accelerometers, magnetometers, and gyroscopes, which are built into the smartphone. After pressing the start button on the Matlab application, the participants repeatedly performed pre-defined motions (e.g., running, walking), and after the motions ended, the signal data was automatically uploaded to a cloud server. We then downloaded the signal data and followed our procedure to predict the user’s motions. For discriminating the walking and running motions in our experiments, we refer to the definitions [49] that one foot is always on the ground while walking, while both feet might simultaneously should be off the ground during running. The whole process of obtaining sensor signal data from the smartphone is shown in Figure 6.

Also, the detailed procedure for obtaining the data is as follows:

(a) Set the predetermined route for motions to be collected for the data (e.g., walking, running).
(b) Place the smartphone on the thigh and tied it up to prevent shaking.
(c) Execute the Matlab mobile application on the smartphone.
(d) Set the sampling rate at 30 Hz, and set it to upload its sensor log to cloud storage.
(e) Select the angular velocity sensor (among the acceleration, magnetic field, orientation, angular velocity, position sensor).
(f) Press the start button to begin acquiring sensor data in the Matlab application.
(g) The participants perform the predefined motion 3 s after pressing the start button to eliminate any effects that may have occurred before executing the action.
(h) The participants perform the motion for about 60 s, which could total 1800 samples.
(i) The participants ceases the motion and presses the stop button 3 s afterward, for the same reason of preventing noise related problems.
(j) After identifying and naming the data set, the data are uploaded to the cloud server.
(k) Download the data acquired from the gyro sensor to a desktop computer.
(l) Repeat steps (d) to (k) for other motions.

The acquired signal consists of three axes of the gyro sensor, the x-, y-, and z-axes. Various methods for treating the x, y, and z axes for sensor data in recurrence plots have been introduced. In this paper, we utilized the strategy of concatenating the x, y, and z axes by referring to [22], where in the process of creating recurrence plots for the x, y, and z axes from a sensor, they first create recurrence plots for each axis and then concatenate the generated recurrence plots side by side. Although the order is slightly different, our approach follows essentially the same process in obtaining the recurrence plots. More precisely, we first create the X vector by concatenating the x, y, and z axes from a sensor and then create the recurrence plots for the X vector. We empirically found that the method of aligning the x, y, and z to construct the X vector and thereby creating the recurrence plot shows satisfactory performance. Let $\omega_{x}$, $\omega_{y}$, and $\omega_{z}$ be the raw sensor signals associated with each axis obtained from the smartphone sensor. We split the time sequence of each axis by using a time window for size, $T_{w}$, and stride *s*, and then combine the split windows on each axis as follows:

(6) $$X_{t}=\left[\omega_{t-5}^{x},\omega_{t-4}^{x},\ldots ,\omega_{t}^{x},\omega_{t-5}^{y},\omega_{t-4}^{y},\ldots ,\omega_{t}^{y},\omega_{t-5}^{z},\omega_{t-4}^{z},\ldots ,\omega_{t}^{z}\right]$$

We converted the raw sensor signal to a recurrence plot with $X_{t}$. In the experiments, we set the window size and stride as 5 and 1, respectively. Thus, the sensor signals are arranged in a dimensionality of 15 (=3 axis × 5 time windows), and we additionally performed linear interpolation on the $X_{t}$ with the Scipy.interpolation [50]. Note that when the frequencies of the input data are not similarly fixed, we need the capability of interpolation. For interpolation, we inserted one additional sample between the original samples, which are obtained from the sensor via linear interpolation until the last sample. After the interpolation, we transformed $X_{t}$ into recurrence plot data according to Equation (3).

(7) $$Y_{t}=Recurrence\left(X_{t}\right)\;\mathrm{where}\;X_{t}\in R^{29}$$

**Figure 7:**
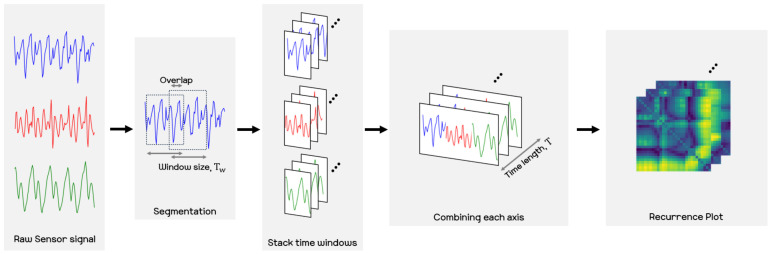
Procedure for converting raw sensor signals into a recurrence plot. Raw sensor signals are divided into time windows of size $T_{w}$ and then converted into a recurrence plot by combining time windows corresponding to each x-, y-, and z-axis into one.

**Figure 8:**
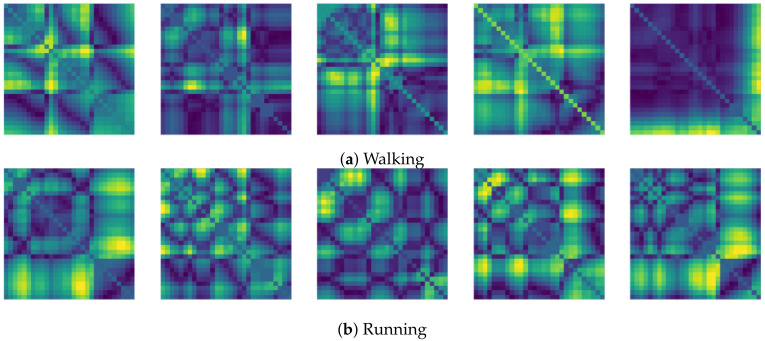
Recurrence plot samples for each motion from the gyro sensor.

The final resolution of the recurrence plot data is then 29×29. In our experiments, we then attempted to predict the next few seconds from the FNO model by receiving the recurrence plot data for the past few seconds. We set the input time step, *T*, and the prediction time step, $\hat{T}$, as 30 and 20, respectively. That is, when the recurrence plot corresponding to 1 s is given as $Y_{t-30:t}$, the FNO model predicts samples, $Y_{t:t+20}$, associated with about 0.6 s of the recurrence plot. In the training phase, the 30 recurrence plots serve as an input to the FNO model, and the following 20 recurrence plots are targets that the model should predict. The whole process for producing the recurrence plot used in this paper is shown in Figure 7 and Procedure 2. Also, Figure 8 shows samples of the recurrence plot created by following the process of Procedure 2 for the motions considered in the experiment.
**Procedure 2**: Procedure for Obtaining Recurrence Plot1:Set parameters window size $T_{w}$.2:Acquire raw sensor signals from a wearable device (e.g., gyroscope): $\omega^{x},\omega^{y},\omega^{z}$.  3:Set empty list to stack time window of each axis, $D_{x},D_{y},D_{z}$.4:**for**$t=T_{w},\ldots ,length\left(\omega \right)$**do**▹ Stack the time windows of each axis5:    $D^{x}\leftarrow D^{x}\cup \left\{\left[\omega_{t-T_{w}}^{x},\omega_{t-(T_{w}-1)}^{x},\ldots ,\omega_{t}^{x}\right]\right\}$6:    $D^{y}\leftarrow D^{y}\cup \left\{\left[\omega_{t-T_{w}}^{y},\omega_{t-(T_{w}-1)}^{y},\ldots ,\omega_{t}^{y}\right]\right\}$7:    $D^{z}\leftarrow D^{z}\cup \left\{\left[\omega_{t-T_{w}}^{z},\omega_{t-(T_{w}-1)}^{z},\ldots ,\omega_{t}^{z}\right]\right\}$8:**end for**  9:Join split time window of each axis.10:**for**t=0,…,length(*D*) **do**11:    $X\leftarrow X\cup \{\left[D_{t}^{x};D_{t}^{y};D_{t}^{z}\right]\}$12:**end for**13:Interpolate for each $X_{t}$.  14:Calculate recurrence plot with $X_{t}$ interpolated through Equation (3).15:**for**t=0,…,length(*X*) **do**16:    $Y_{t}=Recurrence\left(X_{t}\right)$17:**end for**

In this paper, we introduce a method for predicting human motion signals using modern deep learning techniques and smartphone sensors. For this we deal with the two types of input data, for the FNO model and for the decoder network. The FNO model is trained with the recurrence plot data, which are converted from the raw sensor signals, and the decoder model is trained with both the recurrence plot and raw sensor signals to recover the raw sensor signals from the recurrence plot. Given $Y_{t-30:t}$, the input data consisting of the recurrence plot, the FNO model provides the predicted outputs, $Y_{t:t+20}$, which are also the recurrence plot type. We recover the recurrence plot data for the sensor signal type by means of the decoder network. The output of the decoder model would be then the signals, $X_{t:t+20}$, which follow the input signals corresponding to the input of the FNO model.

### 3.2. Experimental Results

**Figure 9:**
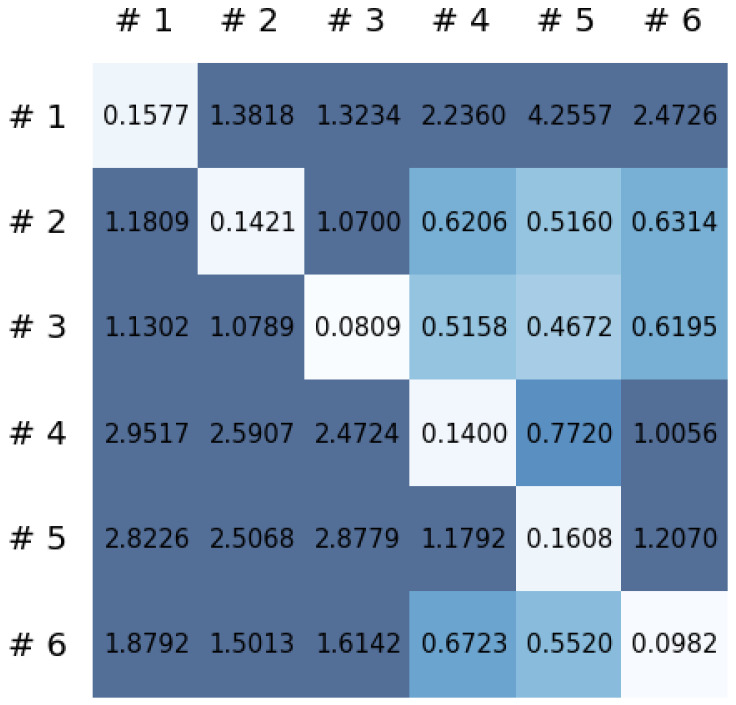
Mean squared error values of the FNO model for each subject for walking motions.

**Figure 10:**
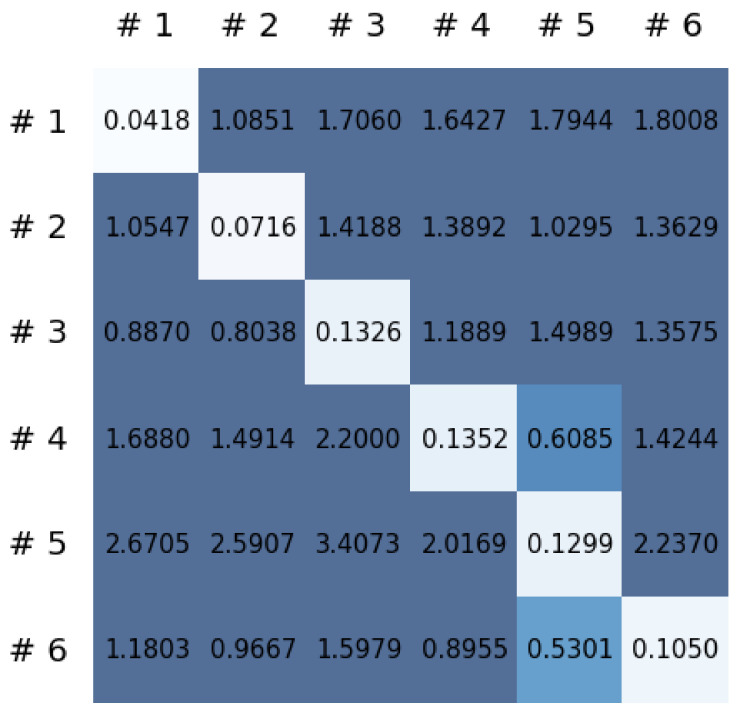
Mean squared error values of the FNO model for each subject for running motions.

This section reports on the experimental results of the proposed method for predicting human motion signals. After recurrence plot data are converted from the raw gyro sensor signals via the process described in Section 3.1, we use the FNO model to predict the upcoming motion signals. To verify the results of the FNO model from the recurrence plot data, we collected motion data from six volunteers, and each volunteer performed the experiment with walking and running motions. The sensor signals were collected for about 60 s at 30 Hz and converted into the recurrence plot data, and then the converted data are entered as input into the FNO model, as described in Section 2.2. Through this process, we can obtain an estimated event sequence for a motion type based on the recurrence plot and FNO model, and then the data are converted into sensor signal form by the decoder model. In the training phase, we set the batch size and epoch at 32 and 200, respectively, and the number of Fourier neural layers in the FNO model was set at 4. The FNO model for recurrence plot prediction and the decoder for converting images into sensor signals are trained separately by means of the Adam optimizer [51]. We also use a learning rate schedule, such that the learning rate starts at 0.001 and decreases by half every 50 epochs. The mean squared error (MSE) loss is used as the loss to training both models. For training and analyzing the experimental results, we split the entire data set acquired from each volunteer into training, validation, and test sets, with a ratio of 6:2:2, respectively. In the data collection step, each motion was performed for about 1 min per subject, and the samples acquired by the gyroscope at this time are 1800 (30 Hz × 60 s). In addition, the data were collected twice for each motion per subject, and therefore, the total number of samples acquired is about 3600 per subject. We split these data in a 6:2:2 ratio, and the number of training, validation, and test recurrence plots then amount to about 2160, 720, and 720, respectively, per subject. When training the model, only the training set was used, and the validation set was used to check whether the model was overfitted. Figure 9 shows the MSE values of the FNO model for the test dataset that was conducted with walking motion. As noted above, we conducted the experiments with six subjects and trained the FNO model for each subject, so six models were trained. As seen in Figure 9, we can confirm that a personalized model was created for each subject. When tested with each individual test set, which was not used in the training phase, the MSE value is small, but when tested with other participants’ data, the error value is relatively high. Thus, it can be seen that models trained on each individual subject can identify the subject. Also, Figure 10 shows the MSE values for running motion. The results for running motion also show that the models properly characterized each subject.

**Figure 11:**
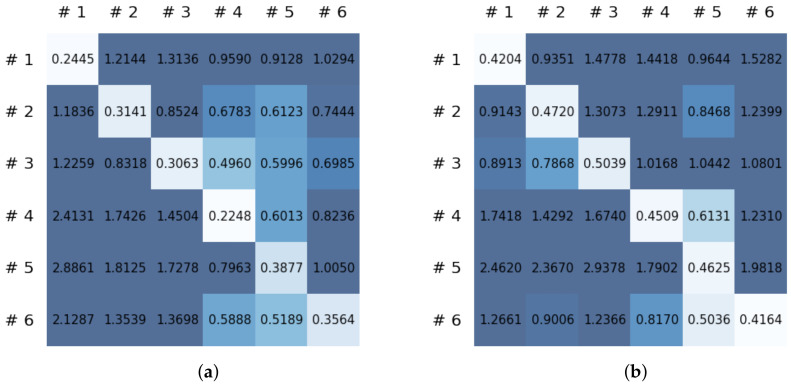
MSE values of the CNN model for (**a**) walking motions and (**b**) for running motions.

**Figure 12:**
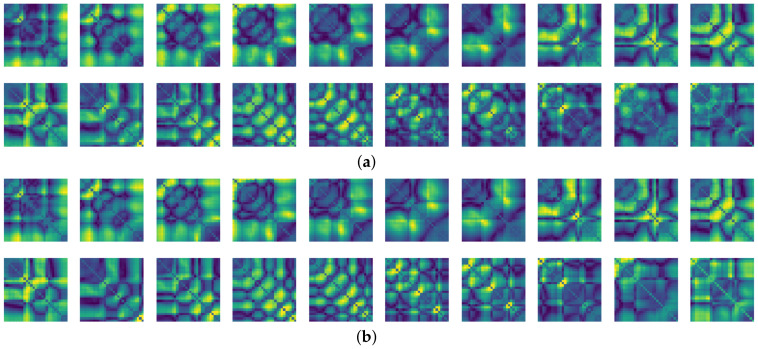

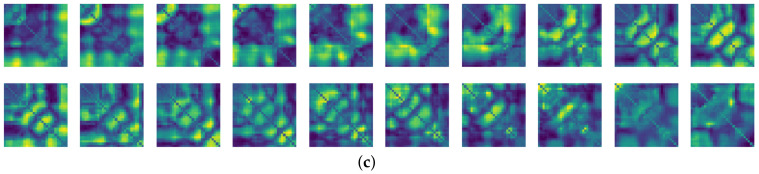
Some samples for walking motion: (**a**) predicted results of the FNO model, (**b**) target recurrence plot data, and (**c**) predicted results of the CNN model.

**Figure 13:**
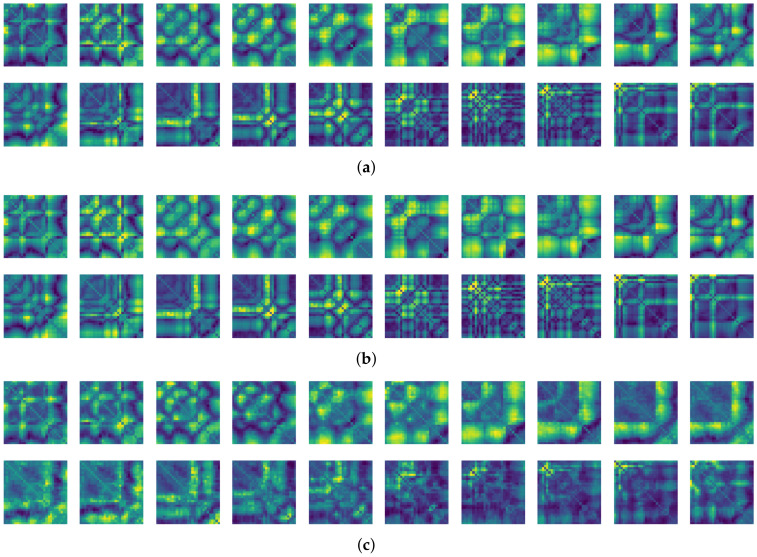
Some samples for running motion: (**a**) predicted results of the FNO model, (**b**) the target recurrence plot data, and (**c**) predicted results of the CNN model.

**Figure 14:**
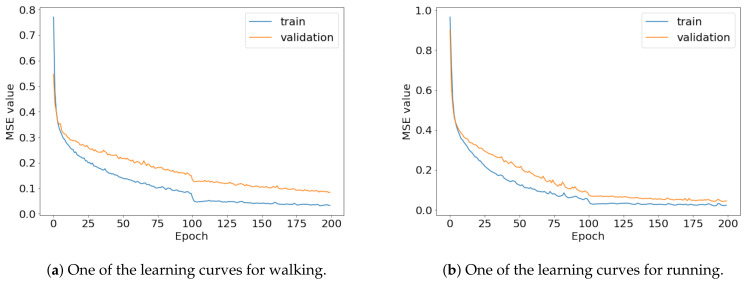
Some of the learning curves of the trained FNO model for (**a**) walking and (**b**) running.

**Figure 15:**
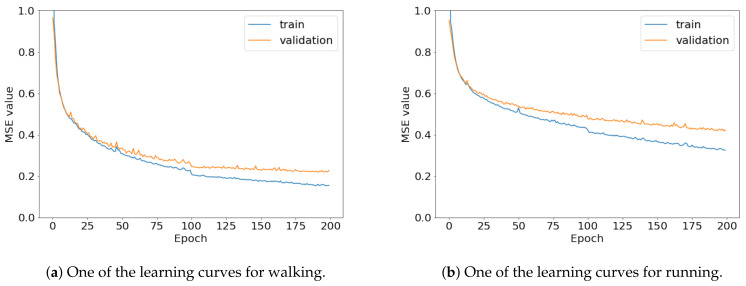
Some of the learning curves of the trained CNN model for (**a**) walking and (**b**) running.

**Figure 16:**
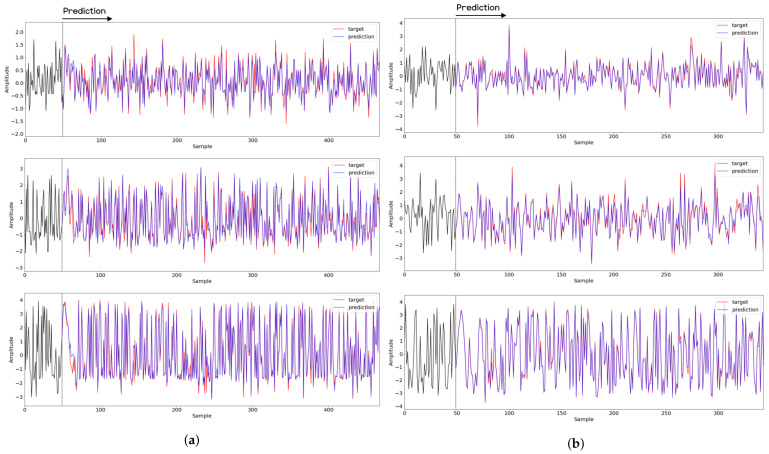
Recovery results for decoder network for (**a**) walking motion and (**b**) running motion. The top, middle, and bottom rows represent the x-, y-, and z-axis, respectively. The blue and red lines indicate the predicted signal from the FNO model and target signal, respectively, and the black line represents the signal corresponding to the input entered into the FNO model.

To compare the results of the FNO model, we trained another model, which is composed of convolutional neural layers, referred to as the CNN model for convenience in the rest of this paper. The CNN model consists of four convolutional neural layers and three fully connected layers, constructed in a manner similar to the FNO model. The kernel size and stride of each convolution layer in the CNN model are 5 and 1, respectively, and zero padding was added so that the image size would not change while passing through the convolutional neural layers. Each convolution layer was followed by a ReLU activation function, except for the final layer. That is, according to the FNO structure, the experiment was conducted by replacing the Fourier neural layer with the convolutional neural layer, while all other layers were the same. With the same learning parameters, optimizer, and learning rate scheduler as in the FNO model, the CNN model was trained. The results of the CNN model in Figure 11 indicate that the CNN model had higher MSE values than the FNO model for the same dataset. For example, the first diagonal entries of Figure 9 and Figure 11a show that the MSE values of the FNO model and CNN model for subject #1 are 0.1577 and 0.2445, respectively. Furthermore, the first diagonal entries of Figure 10 and Figure 11b show that the MSE values of the FNO model and CNN model for subject #1 are 0.1421 and 0.3141, respectively. As shown in Figure 9, Figure 10 and Figure 11, the MSE values of the CNN model turn out to be higher than those of the FNO model throughout all subjects. From these results, we can confirm that the FNO model can better represent human motions expressed by converting sensor signals into an image format than a simple CNN model. Figure 12a and Figure 13a show some samples of the predicted results of the FNO model for walking and running motions, respectively. These figures show that the predicted results almost equal the target recurrence plot images, in Figure 12b and Figure 13b. On the other hand, in Figure 12c and Figure 13c, in comparing the results with the FNO model, some of the CNN model samples are also shown, and those CNN results shown lower predictive performance. With these results, the validity of the method for predicting human motion using the FNO model can be empirically confirmed. We additionally utilized the learning curves for the training and validation dataset during the training process to check whether the model overfits. Figure 14 and Figure 15 show some of the learning curves of the model trained for each subject. One can see that if the loss value for the validation dataset stops improving at some point and the loss value begin to increase, then it can be considered overfitting. Our learning curves, Figure 14 and Figure 15, show that when training the model, the results do not suffer from overfitting. Furthermore, as mentioned in the previous Section 2.3, we also use the decoder network to reconstruct from the recurrence plot images to the sensor signal. After predicting the recurrence plot from the FNO model, we recover the sensor signal corresponding to the predicted recurrence plot image by using the decoder. Figure 16 shows the reconstructed sensor signal utilizing the trained decoder model after obtaining the predicted recurrence plot from the FNO model. We then plot some of the subjects’ results and all the prediction results, but only a portion of the input is displayed. Since the prediction length of the FNO model is set to 20 time steps, the output of the decoder is also 20. However, for simplicity, we aggregate all prediction results except for duplicates and plot them at once, while the input signal plots only portions of them. Figure 16a shows the results for walking motion, indicating the x-, y-, and z-axis sequentially from the first row. The results show that despite image data type of the recurrence plot, the conversion results from the decoder work well. Similarly, the decoder’s output for running motion in Figure 16b also shows that it has well recovered the sensor signal, and thanks to the satisfactory prediction results of the FNO model, it does not differ much from the actual target signals. Note that in this paper, the objective is constructing a personalized model for each subject by means of the modern deep learning model, the Fourier neural operator, and recurrence plots. The model presented in our experiments might be viewed as not generalized. However, we did not aim to make a generalized model that could predict the movement of various individuals with one model. A personalized model capable of predicting the movement of a specific person was considered in this paper. It is expected that this personalized model could be utilized not only to predict movements related to daily activities, but also to provide customized support such as exercise programs and physical therapy, which could lead to a wide range of health benefits.

## 4. Discussion and Conclusions

### 4.1. Discussion

**Figure 17:**
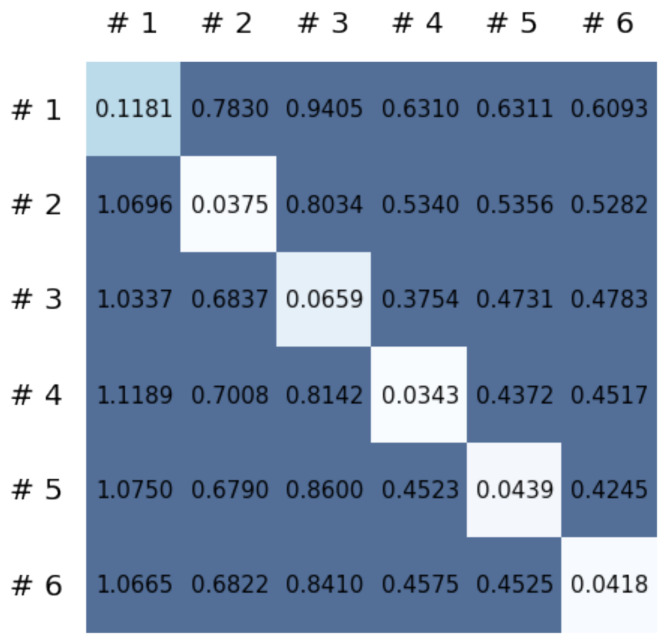
MSE values of the FNO model using CRP for each subject for walking motion.

**Figure 18:**
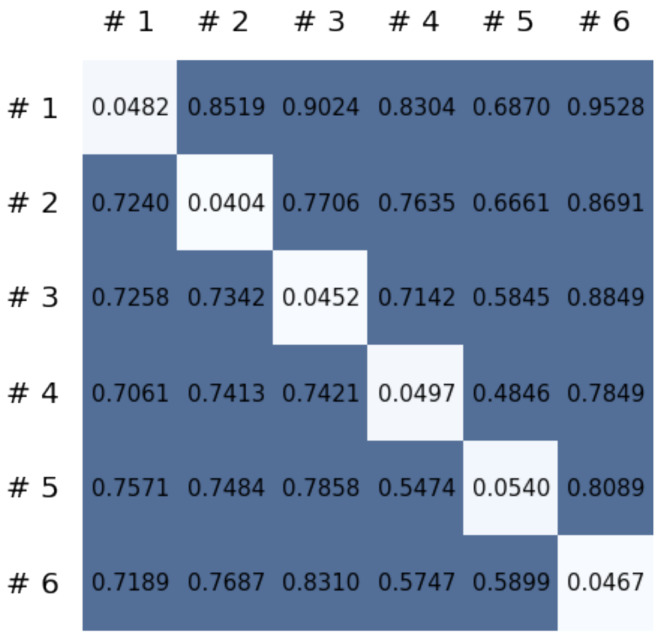
MSE values of the FNO model using CRP for each subject for running motion.

**Figure 19:**
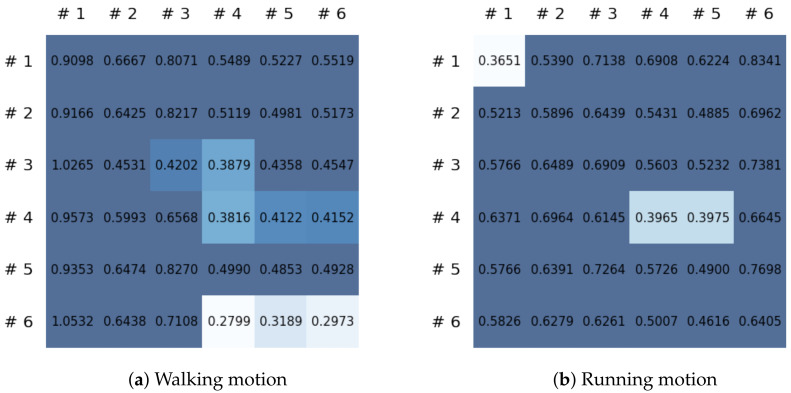
MSE values of the CNN model using CRP for each motion.

**Figure 20:**
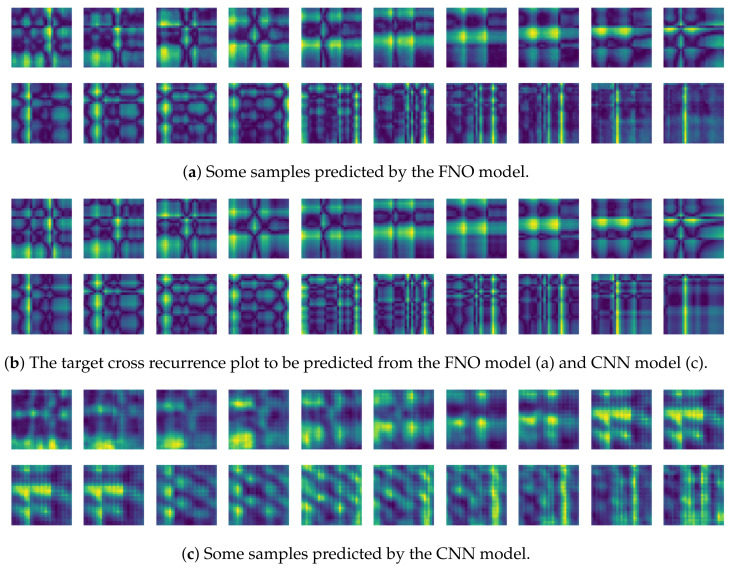
Some samples for walking motion: (**a**) predicted results of the FNO model, (**b**) target cross recurrence plot, and (**c**) predicted results of the CNN model.

**Figure 21:**
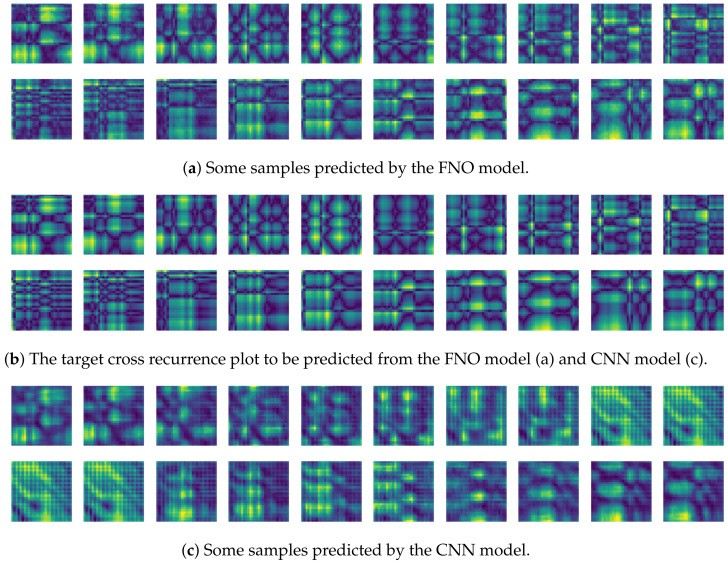
Some samples for running motion: (**a**) predicted results of the FNO model, (**b**) target cross recurrence plot, and (**c**) predicted results of the CNN model.

**Figure 22:**
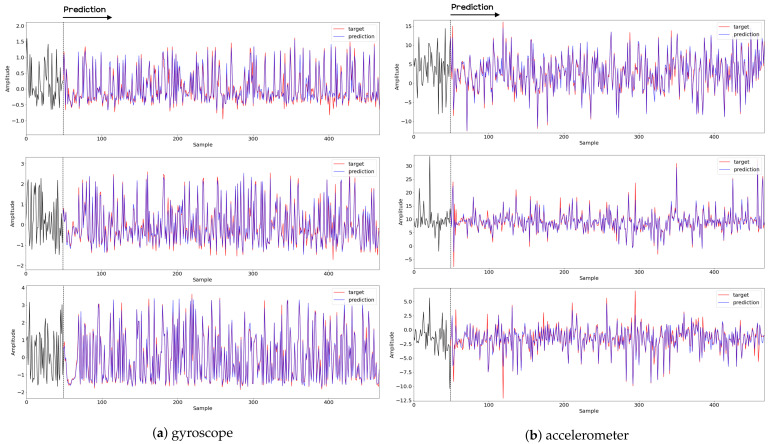
Samples of recovery results from the decoder model for walking motion. The top, middle, and bottom rows represent the x-, y-, and z-axis, respectively. The blue and red lines indicate the predicted signal from the FNO model and target signal, respectively, and the black line represents the signal corresponding to the input entered into the FNO model.

**Figure 23:**
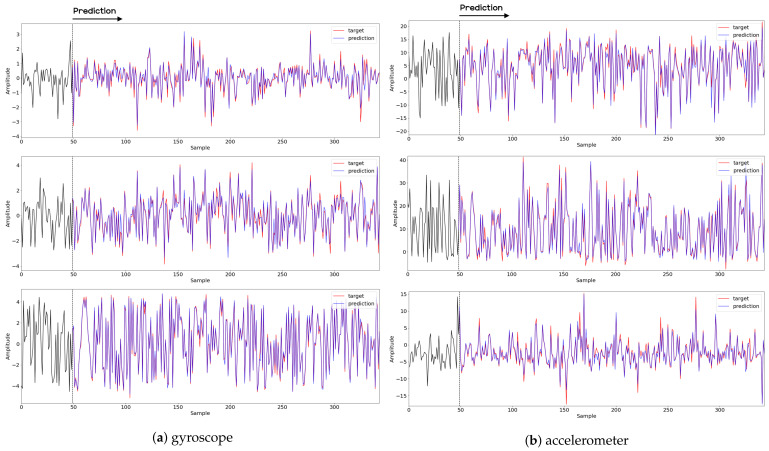
Samples of recovery results from the decoder model for running motion. The top, middle, and bottom rows represent the x-, y-, and z-axis, respectively. The blue and red lines indicate the predicted signal from the FNO model and target signal, respectively, and the black line represents the signal corresponding to the input entered into the FNO model.

In this paper, a study was conducted to predict sensor signals for human motions (e.g., walking and running) using gyro sensors, recurrence plots, and a recent deep learning model, referred to as a Fourier neural operator (FNO). Our applications of FNO along with recurrence plots should be timely and meaningful. Activities such as walking and running are regular and somewhat periodic, but it is also true that they are indeed the most important components in daily life activities. Since methods utilizing frequency domain information are generally efficient in dealing with periodic (or quasi-periodic) signals, we believe that the use of a Fourier neural operator, a modern innovative frequency domain approach, in characterizing daily activities merits investigation. Through the results of this paper, we show that when 30 samples, which are equal to 1 s of signals, were entered as input into the FNO model, prediction results were confirmed to be accurate for the next 20 samples, or about 0.6 s. The sensor signal is obtained from the gyroscope which is built in a smartphone, which is described in Section 3.1, and the data are converted into the recurrence plot to input into the FNO model, as described in Section 2.1. In the main experiment of this paper, we used only the gyroscope data. Smartphones contain various sensors, such as accelerometers, magnetometers, and gyroscopes, and we also consider using gyroscope and accelerometer data in this section. The procedure is the same as in the previous main experiment, but we consider replacing a section of the recurrence plot consisting of gyroscope data with data obtained from both the gyroscope and accelerometer. Referring to a previous study on creating recurrence plots from two sensors [52], the recurrence plot used in our main experiment is replaced by the cross recurrence plot, or CRP, as described in Section 2.1. Figure 17 and Figure 18 show the MSE values for the test data for walking and running motions, respectively, when the FNO model was trained with the CRP. As seen in the results, even when the gyroscope and accelerometer data were used together, it was capable of creating personalized models for each subject. After models for each subject are trained with their own data, they show low MSE values when their own test data (i.e., data not used for training) are provided in the model, but show high MSE values when the test data of another subject is entered. On the other hand, the CNN model shows overall high MSE values (as seen in Figure 19), and it can be seen that the predictive model construction has failed as well as the personalized model. Not only the quantitative analysis of MSE values, but also the qualitative results from the cross recurrence plots predicted from the FNO model can be confirmed, as seen in Figure 20 and Figure 21. Figure 20 is for walking motion, where the first row shows the predictive results of the FNO model, the second row shows the target cross recurrence plot that should be predicted from the FNO and CNN models, and the last row shows the outcomes predicted by the CNN model. These results indicate that the predicted cross recurrence plots by the FNO model are quite similar to the target, but those from the CNN model show differ from the target. Similarly, Figure 21 shows the predictive results of the FNO model for running motion, the target CRPs, and the predictive results of the CNN model. Similar to the walking motion results, this shows that the FNO model predicted the target well, but the CNN model did not. Figure 22 and Figure 23 represent reconstructed sensor signals using the predicted CRP from the FNO model for walking and running motions, respectively. Since the gyroscope and the accelerometer are used in combination, the recovered sensor signals from the CRP also have two signals related to each sensor. In each figure, (a) represents the x, y, and z axes of the gyroscope in order from the top, and, similarly, (b) represents the x, y, and z axes of the accelerometer. Although it could be a rather difficult process to recover the signals from the CRP, in which information from the two sensors is combined, it can be seen that the recovered sensor signals via the decoder model are similar to the target signals.

**Figure 24:**
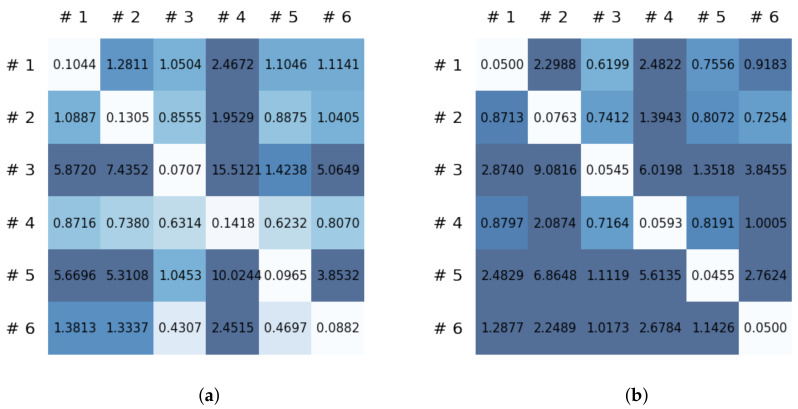
MSE values of the FNO model for (**a**) walking down stairs and (**b**) for walking up stairs.

**Figure 25:**
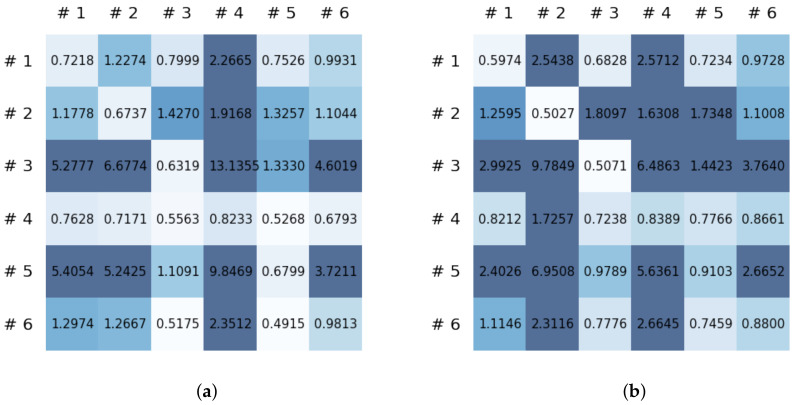
MSE values of the CNN model for (**a**) walking down stairs and (**b**) for walking up stairs.

We also considered walking up and down stairs, and for this experiment, we utilized a public dataset, MotionSense [41]. As mentioned in [41], the dataset was collected at 50 Hz, and an iPhone 6s was used as the smartphone device. The smartphone was located in the front trousers pocket. We conducted numerical experiments for the MotionSense dataset in a manner similar to our previous experiments. Figure 24 and Figure 25 show the results of the FNO model and the CNN model, respectively. As shown in the figures, the MSE performance of the FNO model turns out to be better than for the CNN model, which shows that models trained on each individual subject can identify the subject.

One may have concerns that the presented approach lacks completeness in its human movement analysis. However, the main point of this paper is not to cover complete human movements, but to present a methodology for personalized healthcare. In this paper, we covered walking and running motions, which are the most common activities people perform in their daily lives, and this can be expanded to a variety of motions that comprise daily activities. Further study will be conducted, but we think that an expansion in scope would not be unreasonable because the FNO model also worked well for additional motions, such as walking up and down stairs. We expected that if further research like this is conducted, possible future applications would include personalized healthcare, such as assistance in physical exercise and rehabilitation therapy.

**Figure 26:**
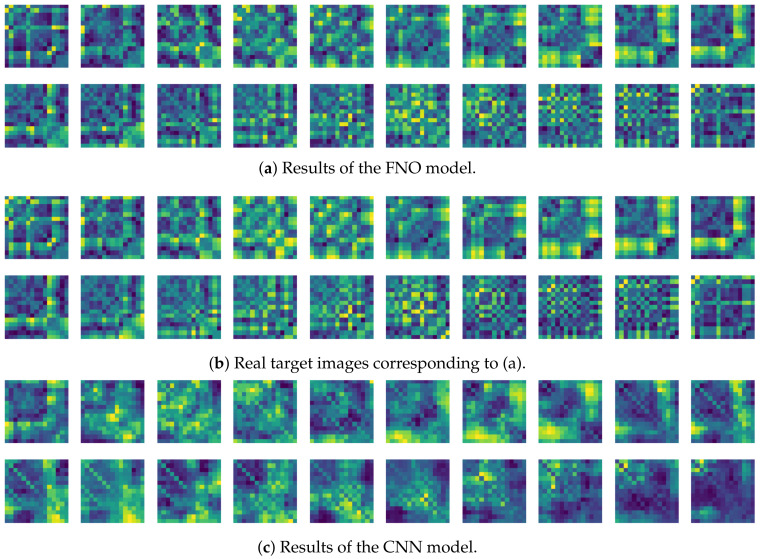
Some samples for running motion.

**Mesh Invariance.** As discussed above and in previous research, FNO has the advantage of being mesh-independent. In order to confirm this characteristic of FNO, we first train the FNO model with the recurrence plot image, as introduced in Procedure 2, and the time window was set at 5. After training the FNO model, the trained weights were fixed and the time window corresponding to the image resolution was shortened. The results of the predicted recurrence plot image from varying the resolution of the recurrence plot are shown in Figure 26. As seen in the figure, the FNO model results are similar to the target recurrence plot images, but the results of the CNN model appear to differ from the target. From these results, we can experimentally check the mesh-free character of the Fourier neural operator [30].

**Fall Detection.** According to the World Health Organization(WHO), falls are the second leading cause of death in the world, and are particularly fatal to adults over the age of 60 [53]. For these reasons, fall detection has become a major research field in the health area and the topic of many studies [54,55,56]. The results of this study confirm that human motion signals are well predicted using the FNO model and a gyro sensor. Accordingly, we consider fall detection by means of the FNO model and modern energy-based methods [57] for distinguishing abnormal movements corresponding to potential falls from normal motion. The energy-based method is particularly attractive, in that it does not require learning new parameters. Additionally, as introduced in one paper [57], the energy calculated from logit values makes for a clearer distinction between distributions of normal and abnormal movement than softmax, which is generally used for classification. Thus, if a model is trained to classify motions (e.g., walking and running), we can utilize it to detect falls without introducing or learning any new parameters. The problem of classifying activities of daily living (ADL) has been widely studied, with suitable performance demonstrated using deep learning methods [8,9]. One of the important capacities related to fall detection is to quickly alert guardians or supervisors when a fall occurs involving the elderly or infirm who are vulnerable to falls, and we consider the possible usefulness of the proposed method for this capacity. Following the strategy of the one-class classification approach [58,59], we use the running and walking motion data as normal samples, and treat the motion data in the course of falling as abnormal samples. The model makes a decision about whether a given data sample is to be classified as normal, and when an anomaly occurs, we can quickly inform users of a risk of falling. In this paper, we constructed a simple deep neural network model composed of two fully connected layers, and trained it to classify input data such as walking or running. After training the classifying model, we use the energy computed from the logit values of the last layer and the threshold computed in the training phase to distinguish whether a behavior is normal (i.e., walking and running) or a likely fall. To utilize the successive prediction results of the FNO model, we fed the predicted time step recurrence plots as input into the classifier in the training and inference phase. It can thereby distinguish subsequent motion events predicted by the FNO model as a fall or as normal motion.

**Figure 27:**
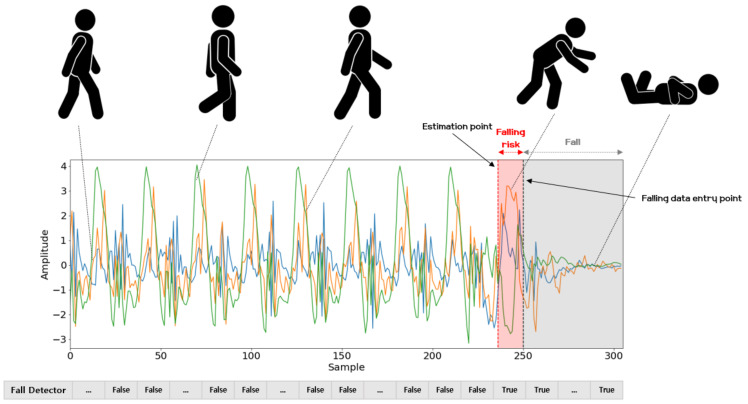
Results of fall detection experiments. The estimation point can be used as a point in time to transmit an indicator that informs the user in advance that a fall may occur when the fall detector detects a potential fall, by means of the FNO predictive results for the next time step data. After that, when actual fall data is entered, it can be viewed as a fall occurrence.

Falling conditions can thus be predicted more quickly than fall detection methods using current observational data. As shown in Figure 27, if a user is about to fall while walking, the FNO model can alert users of a falling risk slightly faster. When the classifier predicts the FNO model prediction results for the next time step as potential falling, it can provide the user an alert that the user may fall, and when actual falling data comes as an input after the falling risk signal, it can be classified and recorded as a fall. Through the results of this experiment on fall detection, it is possible to predict and notify users immediately before an actual fall occurs. Finally note that here, we only examined the potential of the proposed fall detection method for future applications. Therefore, it would be unsuitable for use in fall detection using the proposed method in its current form. In future work, we expect that this could be made more feasible for fall detection by acquiring data on various motions related to daily activities, which could expand the distribution of normal data, after conducting additional research.

**Figure 28:**
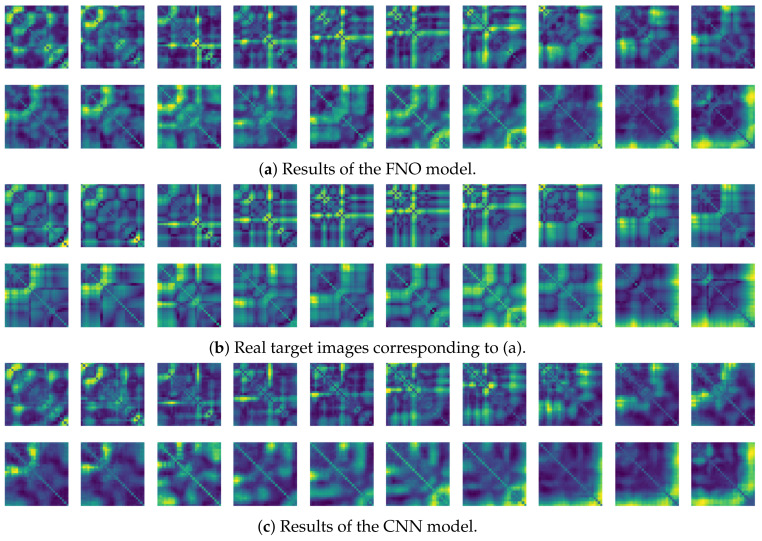
Some samples for walking motion using accelerometer.

**Gyroscope Drift.** Since we use the gyroscope data in the experiments, there may be concern about drift, which is often an obvious challenge in long-term movements. In order to address this problem, an additional experiment was conducted on a subject. The subject performed the same walking motion as in the previous experiment in Section 3, and performed it for 1 min after walking in place for 10 min at normal walking speed. Note that except for this difference (whereby the walking motion was performed after walking in place for 10 min), the experimental procedures and training conditions remain the same. Section (a) and (c) of Figure 28 show the recurrence plot prediction results for the FNO model and CNN model, respectively. As seen in these results, the FNO model turns out to have better predictive performance than the CNN model. In addition, Table 1 shows that the FNO model has lower MSE values compared to the CNN model. These observations indicate that the use of gyroscope data after walking in place for 10 min does not alter the results. Furthermore, we believe that the manner in which Equation (3) deals with gyroscope data, with the norm of relative difference, has less effect on the drift problem.

### 4.2. Conclusions

In this paper, human motion prediction was performed using a gyro sensor and a modern deep learning method, the Fourier neural operator [30]. Predicting human motion has been being widely studied in many fields, with various studies on predicting human behavior in specific domains and environments, e.g., with surveillance cameras, as well as behavior prediction without space restrictions with wearable sensors. In this paper, a study is conducted to predict motion using a commonly used and popular device, specifically, a method using a gyro sensor built into a smartphone. The advantage of using wearable devices such as smartphones is that they can be used without spatial limitations or additional costs. For these reasons, a built-in smartphone gyro sensor and a modern deep learning method are used to analyze human motion signal input from the sensors to predict subsequent signals. Deep learning methods have recently demonstrated suitable performance, and among the various types of research being conducted, we utilize the Fourier neural operator [30] for predicting the next time step of human behavior. In the results for the Fourier neural operator shown in Section 3.2, we checked the performance for predictive ability of the Fourier neural operator. As soon as sensor signal samples corresponding to 1 s are available, the next time step signal for the subsequent 0.6 s is predicted well by the Fourier neural operator. Furthermore, when the widely used convolutional neural network is used to construct and compare the same form for the FNO, we confirmed that the predictive performance for the Fourier neural operator is better than the predictive results from convolution networks. In Section 4.1, we also considered a method for detecting risks such as falling via human motion signals based on the predictive performance of the Fourier neural operator. Before the observed input signals arrive, the subsequent signal was predicted based on the Fourier neural operator, so it would be possible to warn users of potential danger slightly more quickly than when the observed signal is entered into the fall detector. It was thus confirmed that the subsequent signal can be predicted by means of pedestrian behavior signals obtained from a wearable sensor, and by means of the Fourier neural operator model. Furthermore, it was confirmed that when using the signal predicted by the Fourier neural operator, a warning of a fall risk can be provided slightly more quickly than by using only the current motion signal. In light of these findings, if wearable sensors and cloud servers can be used for this in the future, it will be possible to use these as a service to warn people in advance who might be seriously injured in a fall, such as the elderly.

## Figures and Tables

**Table 1:** MSE values of the FNO model and CNN model for long-term walking motion.

Models	FNO	CNN
MSE value	0.134	0.332

## Data Availability

Not applicable.
